# Evaluation of the role of stress in patients with breast cancer and depression by paykel's life event and adaptive neuro‐fuzzy approach

**DOI:** 10.1002/brb3.1570

**Published:** 2020-02-26

**Authors:** Jovana Cvetković, Svetlana Ivanović Kovačevic, Milan Cvetkovic, Slavica Cvetkovic

**Affiliations:** ^1^ Kliničko‐bolnički Centar Gračanica Belgrade Serbia; ^2^ Faculty of Medicine Novi Sad Department of Psychiatry and Psychological Medicine University of Novi Sad Novi Sad Serbia; ^3^ MSc QF at Swiss Federal Institute of Technology & University of Zurich Zurich Switzerland; ^4^ Faculty of Technical Sciences University of Priština Kosovska Mitrovica Serbia

**Keywords:** adaptive neuro‐fuzzy inference system, breast cancer, depressivity, prediction, stress

## Abstract

**Introduction:**

The aim of this study was to identify and analyze the stress factors and the level of stress a year preceding the onset of breast cancer and depression in the studied female patients.

**Methods:**

The research in this work was mostly prospectively (clinical and analytical). During the research, Scale of Life Events‐Paykel was applied. Stressful life events differed significantly between the groups. In the studied group, the following events were significantly more prevalent: partner infidelity (*χ* ^2^ = 12.663; *p* < .001), failure at work (*χ* ^2^ = 44.429; *p* < .001), and spontaneous abortions or stillbirths (*χ* ^2^ = 13.818; *p* < .001).

**Results:**

According to the results of this study, stressful life events differed significantly between the observed groups. These stressful life events had a significant impact on the increase of risk for breast cancer, as well as on depressivity. Afterward, adaptive neuro‐fuzzy inference system was used for prediction of the Paykel's Life Event according to Fisher's exact test.

**Conclusion:**

The obtained results could be of practical usage for improving stress behavior of the patients with breast cancer and depression.

## Graphical abstract

To identify and analyze the stress factors and the level of stress. A year preceding the onset of breast cancer and depression in female patients.

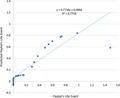

## 1 INTRODUCTION

Stress is a characteristic problem of modern human society, and as such, it should be highly prioritized in the sphere of medical research (Picardi & Gaetano, 2014). Inability to cope successfully with stressful life events leads to maladaptive responses and susceptibility to stress‐associated pathologies.

Our reaction to an acute stress is almost invariably tumultuous. If stressful situations come one after another, our organism does not have enough time to recover adequately (Biondi & Picardi, 1999). Chronic stress is the result of poor adaptive response to a sequence of unpleasant and upsetting long‐term life situations which tend to exhaust human defense mechanisms. Individuals with insufficient (deficient) defense mechanisms in stressful situations tend to react with chronic, prolonged stress, associated with the feeling of depressivity, uneasiness, insidious fear, and with various accompanying neurotic reactions. About one third of the general population, at least once during a lifetime, experience an extreme stress or stressful event.

Breast cancer is a long‐known disease, which with its participation in general morbidity and mortality is nowadays one of the most important health care concerns throughout the world. Facing a malignant disease, involving the diagnosis and often long‐lasting, demanding, and uncertain treatments, represents a huge shock to the patient, stress, and introduction to a serious crisis in life, producing psychic instability. This could all have an unfavorable effect upon the course and outcome of the treatment.

This paper deals with the stress preceding breast cancer and depression. To do so, adaptive neuro‐fuzzy inference system or ANFIS (Gavrilović, Denić, Petković, Živić, & Vujičić, 2018; Jang, 1993; Milovančević et al., 2018; Nikolić, Mitić, Kocić, & Petković, 2017; Petković, 2017) is used which is soft computing technique suitable for strong nonlinear relationship between input and output data pairs. The main goal is to predict Paykel's Life Event according to Fisher's exact test. The main motivation is stress reduction in the initial stage of the breast cancer and depression.

## 2 METHOD

The study enrolled 153 female examinees, out of which 103 (67.3%) with breast cancer comprising the studied group, and 50 (32.0%) women with depression and without breast cancer as the control group. The average age of the whole studied population was 49.20 ± 11.2 years (age range from 26 to 65 years). As for the age of the enrolled examinees, the groups were not statistically significantly different, that is the groups were homogenous (studied group: 49.4 ± 10.8 vs. control group: 48.76 ± 12.21 years; *t* = 0.329; *p* = .734).

The study took place in the outpatient department of the Clinic of Oncology, Clinical Center Niš. The patients were asked to fill in the questionnaires while waiting for their therapy. The Paykel's Life Event scale was used in the study (Kendler, Gardner, & Prescott, 2006; Paykel, 2001; Timptijević & Marković, 1989).

All procedures performed in studies involving human participants were in accordance with the ethical standards of the institutional and/or national research committee and with the 1964 Helsinki Declaration (World Medical Association, 2009) and its later amendments or comparable ethical standards.

Informed consent was obtained from all individual participants involved in the study.

### 2.1 ANFIS methodology

**Figure 1:**
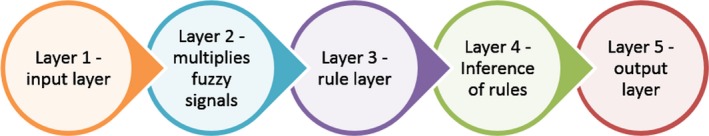
ANFIS layers

ANFIS network has five layers as shown in Figure 1. The main core of the ANFIS network is fuzzy inference system. Layer 1 receives the inputs and converts them in the fuzzy value by membership functions.

Second layer multiplies the fuzzy signals from the first layer and provides the firing strength of rule. The third layer is the rule layers where all signals from the second layer are normalized. The fourth layer provides the inference of rules, and all signals are converted in crisp values. The final layers summarized the all signals and provided the output crisp value.

## 3 RESULTS

Table 1 presents a comparison of significant life events in a year preceding the disease between the observed groups. The following life events were significantly different between the groups: Among those with breast cancer, significantly more prevalent were spouse infidelity (*χ* ^2^ = 12.663; *p* < .001), failure at work (*χ* ^2^ = 44.429; *p* < .001), and spontaneous abortions or stillbirths (*χ* ^2^ = 13.818; *p* < .001).

**Table 1:** Paykel's Life Event scale

		Studied group	Control group	Fisher's Exact test	*p*
Death of a child	No	98 (95.1)	50 (100.0)		
	Yes	5 (4.9)	0 (0.0)	2.509	.113
Death of a spouse	No	96 (93.2)	42 (84.0)		
	Yes	7 (6.8)	8 (16.0)	3.225	.073
Prison sentence	No	99 (96.1)	49 (98.0)		
	Yes	4 (3.9)	1 (2.0)	0.378	.539
Death of a close family member	No	101 (98.1)	31 (62.0)		
	Yes	2 (1.9)	19 (38.0)	36.959	<.001
Spouse infidelity	No	67 (65.0)	46 (92.0)		
	Yes	36 (35.0)	4 (8.0)	12.663	<.001
Serious financial difficulties	No	96 (93.2)	18 (36.0)		
	Yes	7 (6.8)	32 (64.0)	57.994	<.001
Failure at work	No	43 (41.7)	49 (98.0)		
	Yes	60 (58.3)	1 (2.0)	44.429	<.001
Discharge from work	No	100 (97.1)	48 (96.0)		
	Yes	3 (2.9)	2 (4.0)	0.126	.723
Spontaneous abortions or stillbirths	No	79 (76.7)	50 (10.0)		
	Yes	24 (23.3)	0 (0.0)	13.818	<.001
Divorce	No	99 (96.1)	49 (980)		
	Yes	4 (3.9)	1 (2.0)	0.378	.539
Marital separation (due to disputes)	No	97 (94.2)	50 (100.0)		
	Yes	6 (5.8)	0 (0.0)	3.032	.082
Involvement in a court case (for serious violations of the law)	No	101 (98.1)	50 (100.0)		
	Yes	2 (1.9)	0 (0.0)	0.984	.321
Unwanted pregnancy	No	101 (98.1)	49 (98.0)		
	Yes	2 (1.9)	1 (2.0)	0.001	.981
Hospitalization of a family member	No	103 (100.0)	42 (84.0)		
	Yes	0 (0.0)	8 (16.0)	17.389	<.001
Unemployment for a month	No	94 (91.3)	48 (96.0)		
	Yes	9 (8.7)	2 (4.0)	1.132	.287
Death of a close friend	No	101 (98.1)	44 (88.0)		
	Yes	2 (1.8)	6 (12.0)	6.872	.009
Shift to a lower position at work	No	92 (89.3)	49 (98.0)		
	Yes	11 (10.7)	1 (2.0)	3.508	.061
Severe (serious) organic disease	No	101 (98.1)	46 (92.0)		
	Yes	2 (1.9)	4 (8.0)	3.279	.070
Start of a relationship outside marriage	No	95 (92.2)	48 (96.0)		
	Yes	8 (7.8)	2 (4.0)	0.782	.377
Loss of a valuable personal possession	No	100 (97.1)	49 (98.0)		
	Yes	3 (2.9)	1 (2.0)	0.110	.740
Under investigation	No	101 (98.1)	50 (100.0)		
	Yes	2 (1.9)	0 (0.0)	0.984	.321
Failure at education	No	102 (99.0)	50 (100.0)		
	Yes	1 (1.0)	0 (0.0)	0.489	.485
Frequent marital disputes	No	103 (100.0)	46 (92.0)		
	Yes	0 (0.0)	4 (8.0)	8.461	.004
Frequent disputes with one's parents	No	99 (96.1)	50 (100.0)		
	Yes	4 (3.9)	0 (0.0)	1.994	.158
Frequent disputes with a fiance or boyfriend	No	103 (100.0)	49 (98.0)		
	Yes	0 (0.0)	1 (2.0)	2.074	.150
Taking out a huge loan	No	102 (99.0)	50 (100.0)		
	Yes	1 (1.0)	0 (0.0)	0.489	.485
Conscription of one's son	No	99 (96.1)	50 (100.0)		
	Yes	4 (3.9)	0 (0.0)	1.994	.158
Disputes with one's boss or a coworker	No	103 (100.0)	49 (98.0)		
	Yes	0 (0.0)	1 (2.0)	2.074	.150
Disputes with an extended family member/colleague from work	No	103 (100.0)	45 (90.0)		
	Yes	0 (0.0)	5 (10.0)	10.648	.001
Moving to another country	No	99 (96.1)	50 (100.0)		
	Yes	4 (3.9)	0 (0.0)	1.994	.158
Menopause	No	103 (100.0)	48 (96.0)		
	Yes	0 (0.0)	2 (4.0)	4.175	.041
Small financial difficulties	No	100 (97.1)	49 (98.0)		
	Yes	3 (2.9)	1 (2.0)	0.110	.740
Separation from an important person	No	102 (99.0)	50 (100.0)		
	Yes	1 (1.0)	0 (0.0)	0.489	.485
New person in a household	No	101 (98.1)	50 (100.0)		
	Yes	2 (1.9)	0 (0.0)	0.984	.321
Retirement	No	102 (99.0)	50 (100.0)		
	Yes	1 (1.0)	0 (0.0)	0.489	.485
Change of the type of work	No	102 (99.0)	49 (98.0)		
	Yes	1 (1.0)	1 (2.0)	0.276	.599
Break up with a partner	No	101 (98.1)	50 (100.0)		
	Yes	2 (1.9)	0 (0.0)	0.984	.321
Moving to another town	No	102 (99.0)	50 (100.0)		
	Yes	1 (1.0)	0 (0.0)	0.489	.485
Mild organic disease	No	103 (100.0)	48 (96.0)		
	Yes	0 (0.0)	2 (4.0)	4.175	.041
Moving to another place in the same town	No	101 (98.1)	50 (100.0)		
	Yes	2 (1.9)	0 (0.0)	0.984	.321
Childbirth or adoption	No	102 (99.0)	50 (100.0)		
	Yes	1 (1.0)	0 (0.0)	0.489	.485
Start of school education	No	103 (100.0)	49 (98.0)		
	Yes	0 (0.0)	1 (2.0)	2.074	.150
Marriage of a child (with parental consent)	No	103 (100.0)	48 (96.0)		
	Yes	0 (0.0)	2 (4.0)	4.175	.041

In the control group of women, the following life events were significantly more prevalent: death of a close family member (*χ* ^2^ = 36.959; *p* < .001), serious financial difficulties (*χ* ^2^ = 57.994; *p* < .001), hospitalization of a family member (*χ* ^2^ = 17.389; *p* < .001), death of a close friend (*χ* ^2^ = 6.872; *p* < .001), frequent marital disputes (*χ* ^2^ = 8.461; *p* = .004), disputes with an extended family member or a colleague from work (*χ* ^2^ = 10.648; *p* = .001), less serious (mild) organic disease (*χ* ^2^ = 4.175; *p* = .041), and marriage of a child with parental consent (*χ* ^2^ = 4.175; *p* = .041).

### 3.1 ANFIS prediction

**Figure 2:**
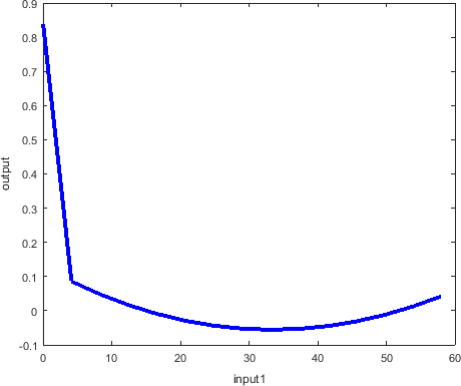
ANFIS relationship between Fisher's exact test (input 1) and Paykel's Life Event (output)

Figure 2 shows obtained ANFIS relationship between Fisher's exact test and Paykel's Life Event. One can note there is strong decreasing of the Paykel's Life Event with increasing of Fisher's exact test in the beginning, and afterward, the Paykel's Life Event is steady.

**Figure 3:**
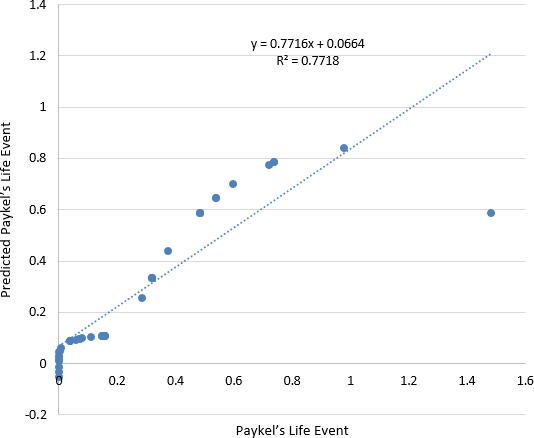
ANFIS prediction of Paykel's Life Event

Figure 3 shows scatter plots of the ANFIS prediction of the Paykel's Life Event based on Fisher's exact test. It can be noted medium predictive accuracy based on *R* ^2^ coefficient. Also, Pearson coefficient (*r*) is .878519 and root mean square error (RMSE) is 0.14903.

## 4 DISCUSSION

In the United Kingdom in the period from 2003 to 2010, a professor at the Institute for Breast Cancer Research investigated whether psychological stress or unfavorable life events had an impact on breast cancer risk (Jemmott & Locke, 1984). The researchers excluded the impact of other risk factors on the results, such as obesity, physical activity, alcohol intake, family history of breast cancer, age at first menarche, age at menopause, number of children, age of their mother at childbirth, duration of breastfeeding, and so on. It was established that 34% of women had reported frequent or continued stress in the 5 years preceding the disease, and 74% had reported at least one unfavorable life event, such as grief (loss or disease of a close person) or divorce. Out of 106,612 women, breast cancer had occurred in 1,783. After the consideration of other risk factors, the researchers established that there was no statistically significant association between the frequency of stress and overall breast cancer risk. Their position was that investigations should be continued, but to involve other possible breast cancer risks as well.

Out of eight characteristic types of stressful events, only one could be demonstrated to have a weak association with the disease. Divorce can be associated with negative breast cancer estrogen receptors in premenopausal women, but that occurred in 25 cases only and had only a statistical importance. This result was not corroborated with increased risk from other similar stressful events, such as grief.

The results demonstrated that women who frequently or continually experienced stress had a risk for breast cancer similar to that in women who never experienced any stress or experienced it only occasionally.

The analysis showed that the risk for breast cancer was slightly higher in women who had lost their mothers early in life, but not if they had lost their fathers. The Center for Chronic Disease Biology studied the issue and showed that stress increased cortisol binding by the glucocorticoid receptor (GR).

According to the results of this study, stressful life events were different between the groups. In the studied group of women, significantly more prevalent were spouse infidelity (*χ* ^2^ = 12.663; *p* < .001), failure at work (*χ* ^2^ = 44.429; *p* < .001), spontaneous abortions or stillbirths (*χ* ^2^ = 13.818; *p* < .001). In the control group, the following life events were significantly more prevalent: death of a close family member (*χ* ^2^ = 36.959; *p* < .001), serious financial difficulties (*χ* ^2^ = 57.994; *p* < .001), hospitalization of a family member (*χ* ^2^ = 17.389; *p* < .001), death of a close friend (*χ* ^2^ = 6.872; *p* < .001), frequent marital disputes (*χ* ^2^ = 8.461; *p* = .004) and disputes with an extended family member or a colleague from work (*χ* ^2^ = 10.648; *p* = .001), menopause (*χ* ^2^ = 4.175; *p* = .041), mild organic disease (*χ* ^2^ = 4.175; *p* = .041), and marriage of a child with parental consent (*χ* ^2^ = 4.175; *p* = .041). These stressful life events had a significant impact on increased breast cancer risk and depressivity (Cooper, Dewe, & O’Driscoll, 2001; Gradus, 2017; Ritter, Antonova, & Mueller, 2012).

There are great individual differences in response even to the same stressors. The authors in one paper (Vlajković, 1992) justly emphasized that stress was a highly personalized process, that is the process highly dependent on personality traits.

Numerous investigations have documented that the status of our psyche has an impact on our health. Cancer occurs only when a human defense system is overpowered and cannot cope with various threats. In a cancer treatment, a significant accent is put on human will, wish, and decision to undergo treatment (Lazarus, 1991).

Our immune system is suppressed by various psychic factors, out of which the most important are long‐lasting grief, feeling of failure in life, anger, anxiety, and stress. Chronic exposure to conflict situations, especially when there is a conflict between our needs, wishes and abilities, creates chronic frustration. When it can no longer be dealt with using the usual defense mechanisms (rationalization, projections, sublimation), a physical disease occurs. Cancer most commonly affects depressive individuals, too rational, afraid of their emotions, with poor fantasies, psychologically restrained and passive (Polansky, 2003).

Numerous studies have shown that stressogenic life events have an impact on the course of depression as well, the quality of remission, and frequency of relapses (Haberstick et al., 2016; Polansky & Javaherian, 2015).

However, the etiological relevance of these facts has not yet been sufficiently elucidated (Paykel, Prusoff, & Uhlenhuth, 1971). For instance, although most depressive patients experience stressful life events before the episodes of depression, only a small portion of individuals exposed to such stressors become actually depressive.

Nowadays, it is a prevalent opinion that a wide spectrum of biological, social, and psychological etiological factors has a role in the genesis of depression. They act upon every individual in different proportions. Except for stressful life events and genetic factors, the authors most commonly mention abuse in early childhood, early loss of parents, quality of social support, etc. (Braš, 2009; Van Praag, Kloet, & Os, 2004).

In some of the investigated clusters, patients have experienced a significantly greater number of stressful life events compared to the control group. Regarding the type of stressors preceding an episode of depression, our results generally agree with the literature data (Brown, Bifulco, & Harris, 1987; May et al., 2008), but there are some differences in their frequency. In our sample, the stressors associated with poor economical situation, unemployment, and aggravation of working conditions are the ones more commonly observed, as expected, in view of the ongoing social transition processes in the country. The frequency of individual stressful life events associated with disturbed partner relationships agrees with the available literature data (Ayuso‐Mateos et al., 2001; Kessler, 1997).

It has been suggested that stress and depression exert similar hormonal and immunological effects (Kessler, 1997), and that stress which often precedes depression is characterized by still unexplained pathophysiological mechanisms.

## 5 CONCLUSION

The aim of our study was to investigate stressogenic life events preceding breast cancer and depression. In the studied patients, a significantly greater number of stressful life events was reported in the year preceding the first and recurrent episodes of depression compared to controls (*p* < .05), which agrees with most other authors’ results. These results confirmed the hypothesis that significant life events in a larger proportion precede the onset of breast cancer than major depression. Afterward, adaptive neuro‐fuzzy inference system (ANFIS) was used for prediction of Paykel's Life Event according to Fisher's exact test. The obtained results could be of practical usage for improving stress behavior of the patients with breast cancer and depression.

## CONFLICT OF INTEREST

There is no conflict of interest.

## AUTHOR CONTRIBUTION

Jovana Cvetković did measurement, Svetlana Ivanović Kovačevic did analyzing, Milan Cvetkovic did analysis, and Slavica Cvetkovic did simulation.

## Data Availability

The data that support the findings of this study are available from the corresponding author upon reasonable request.
